# Migration-induced transition in social structures: a view through the Sakoda model of social interactions

**DOI:** 10.1038/s41598-020-74805-3

**Published:** 2020-10-27

**Authors:** Eric Goles, Aldo Mascareño, Pablo Medina, Sergio Rica

**Affiliations:** 1 Facultad de Ingeniería y Ciencias, Universidad Adolfo Ibáñez, Avda. Diagonal las Torres 2640, Peñalolén, Santiago, Chile; 2 Centro de Estudios Públicos, Monseñor Sótero Sanz 162, Providencia, Santiago, Chile; 3 Departamento de Física, Facultad de Ciencias, Universidad de Chile, Las Palmeras 3425, Ñuñoa, Santiago, Chile; 4 CeiBA Complexity Research Center, Carrera 13A#29-24, Bogotá, Colombia

**Keywords:** Phase transitions and critical phenomena, Complex networks

## Abstract

We study the dynamics of three populations evolving in a two-dimensional discrete grid according to rules of attraction, rejection, or indifference following the framework of the seminal model by Sakoda and we apply it to migration phenomena. An interesting feature of the Sakoda model is the existence of a Potts-like energy which, as a common principle, decreases as time passes by. Here we consider the evolution of two populations until stabilization, then, we perturb this attractor by the inclusion of a third arrival: the immigrants. We show the conditions under which this irruption does not alter significantly the previous attractor (a sociological morphostatic behaviour) or it is dramatically changed (morphogenetic behaviour). We observe empirically that for a morphostatic behaviour the energy decreases while for morphogenesis this energy increases, revealing an escalation of social tension.

## Introduction

In 2015, near to one million migrants and refugees crossed into Europe, mostly coming from Syria, Afghanistan and Iraq. Less massive yet relevant, in 2018 thousands of people literally walked through Central America from Honduras to USA in a series of migration flows. Similar situations took place in Colombia and Chile. Drifting apart from war zones or failed States with limited opportunities for social inclusion (access to work, education, health care, among others), migratory flows have increased in the last years, posing relevant challenges for social structures and institutions in the host countries^1–3^. Societies can react to these flows through a number of peculiar behaviors like, segregation, inclusion, or exclusion. As often happens in complex systems, these social structures produce a collective order that arise from simple local interactions. Despite the intricate nature of social interactions, it is remarkable that social behaviors and structures share many common features with a variety of physical systems, in particular, with magnetic systems, kinetic ordering, and surface tension phenomena^4,5^. In this article, we assess the impact of immigration flows on pre-existing social structures by using Sakoda’s model of social interactions.

After World War II, James Sakoda published his Ph.D. dissertation on the social structures emerging from the basic social interactions among individuals^6^. Only 20 years later, in 1971, by adding a numerical simulation in FORTRAN, this work was published in the Journal of Mathematical Sociology^7^. The main goal of Sakoda’s work was to provide a concrete means of portraying social interaction as an ongoing process among members of groups. The resulting patterns of distances among individuals can be interpreted as the social structure resulting from the interacting process. Mainly on the basis of the conceptual work of the mid-twentieth-century social psychologist Kurt Lewin^8^, Sakoda aimed at formalizing social interactions between two groups to identify emergent patterns that he called social structures^9,10^. The principles of the seminal model are simple: two groups with positive, neutral or negative attitudes to one another moving in an $8\times 8$ chessboard (the space of interaction), trying to improve their position according to the value of a field that takes into account the social preferences of the two populations as well as the distance between individuals. Sakoda identified and described eight self-organized social structures (for example, segregation), which was also considered in the seventies by Thomas Schelling in a series of renowned articles^11–13^. While Schelling’s contribution was successful, Sakoda’s contribution was rather forgotten^14^. However, first, the underlying richness of the Sakoda model offers more analytical options than just the dynamics of segregation, thereby opening possibilities to identify new social structures arising from interaction, as recently done by Medina *et al.*^15^. Second, Sakoda’s aim of connecting psychology’s interest in micro phenomena and sociology’s focus on macro levels corresponds with a key distinction in sociological analysis, that between agency and structure, and subsequently, with the discussion whether agency or structure should be the point of departure for theory building^16^. Third, since Sakoda’s model was practically unattended for about 45 years, a set of technical and theoretical developments in computer sciences, physical modeling, and sociological theory can be applied to explore the possibilities of a generalized Sakoda’s model.

Originally, Sakoda considered only two types of individuals belonging to a network, for simplicity, a square and periodic lattice in two dimensions. Any site in the lattice may be occupied by a state; or it could be empty. These populations interact according to specific attitudes of attraction, repulsion, or neutrality and with a strength that depends on the Euclidean distance among sites in the lattice. In general, the interaction may be of a long or short-range type, but, usually, this strength decreases as the separation distance increases. The basic rule of the model consists of the following steps: first, to take a random individual; next, to evaluate its social expectation at all possible empty sites, finally, the individual moves to the position giving the highest “potential” expectation. This procedure is repeated randomly among all possible individuals.

**Figure 1:**
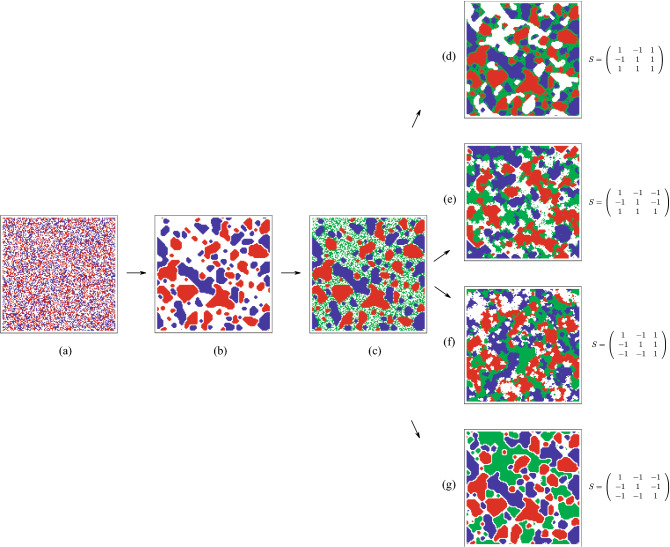
Snapshots of the segregation social structure (S1) process in presence of a third new population. The interaction reads: (  ). (**a**) Shows the initial state with two different populations of (  ,  ) that are randomly and uniformly distributed in the lattice with a concentration  . (**b**) After running or $10^{5}$ steps the system reaches a stationary state. In (**c**) we add a randomly distributed a “green” population with the same concentration  . Next snapshots (**d**–**g**) show the morphogenesis vs. morphostasis after the inclusion of the third population depending on the respective interaction matrix. One notices that the primary segregation structure is not destroyed in the cases (**d**,**g**); however, there are major modifications of the original social structures in the cases (**e**,**f**). We underline the interactions of the case (**g**) corresponds to the three individuals Schelling model: different individuals repel each other but they attract themselves. The simulations evolved in a lattice of $N=128\times 128$ sites and after the inclusion of the “green” individuals the simulation runs for $10^5$ more steps.

**Figure 2:**
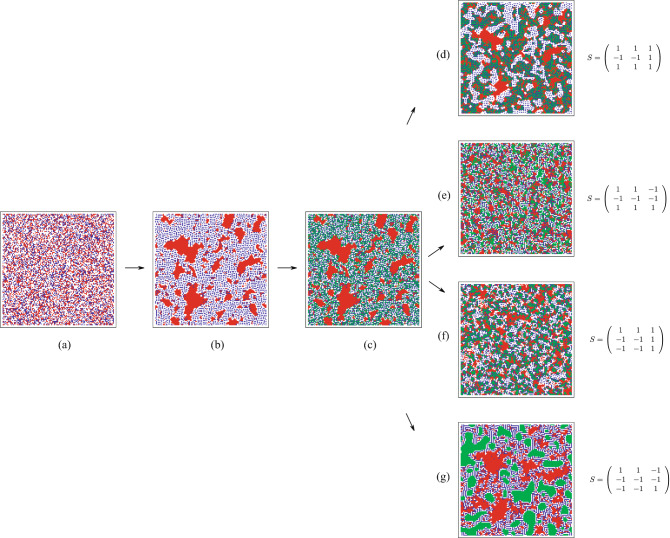
Snapshots of the *Social-workers* social structure (S5) process in presence of a third new population. (**a**) Shows the same initial state as in Fig. 1**a**. (**b**) After running with the corresponding interaction:  , for $10^{5}$ steps reaching a stationary state that consists in a coherent aggregation of red individuals together with a fluctuating populations of “blues” moving eternally. In (**c**) we add a randomly distributed “green” population with the same concentration  . As in Fig. 1, next figures (**d**–**g**) show the morphogenesis vs. morphostasis. One notices that only (**d**,**g**) preserve the primary social structure while other interactions modify substantially the patterns. The simulations evolved in a lattice of $N=128\times 128$ sites.

In Ref.^15^, we have characterized the social structures appearing as a result of the evolution of the original Sakoda model of general social interaction. More important, we quantified the evolution of the 45 different interaction rules via a Potts-like energy function^17^ which drives the system irreversibly to equilibrium or a steady state. From the 45 possible interactions, we have identified 10 basic social structures, the previous seven structures already identified by Sakoda in his Thesis^6^: crossroads, mutual suspicion, segregation, social workers, boys and girls, couples, and husband and wife; and we added in our work three new social behaviors: inclusion, repulsion, and exclusion, which complete the picture. We refer the reader to Ref.^15^ for more detailed description of all social structures. Here we mention a couple of them that we will use explicitly in the developments of our results:

- (S1) *Segregation*, which typifies attraction among individuals of the same group and repulsion among members of the other group. In this case, the system minimizes the interface perimeter inducing segregation (see the forthcoming Fig. 1b).
- (S5) *Social-workers*. In this case, one group feels attraction by everybody while the other group feels repulsion for everybody (see the forthcoming Fig. 2b).

In this article, we apply Sakoda’s social structures model to better understand the dynamics and social consequences of immigration phenomena. Particularly, we aim at identifying the consequences for established social structures when a third immigrant population is introduced in a given social context. This study aims to reflect on these consequences. In order to do this, we develop a three population model following Sakoda’s formulation^7^, evolving on a $L\times L$ board according to a finite set of rules. Then, we study some final social structures for three population obtained through perturbing attractors reached in the Sakoda’s model for two populations; that is, we introduce a third one and let them evolve considering the Sakoda’s approach. We interpret our findings according to Margaret Archer’s sociological approach. In this sense, a society is a continuous interplay between two autonomous levels, agency and structure (as said, the level of interactions among individuals and the macro level of established institutions), whose dynamics evolve in morphostatic/morphogenetic cycles. Roughly speaking, a morphostatic cycle takes place when interactions do not significantly change the functioning of social structures, i.e., when constraints and enablements that social institutions exert upon individuals remain the same—a failed political revolution, for example, does not change and rather reinforce old social structures. On the contrary, in a morphogenetic cycle interaction succeeds in modifying social structures—e.g., passing a new law after a protest movement, the emergence of a new organization after engaged collective action, or a successful political revolution. Applied to our case, a morphostatic transition occurs when the third population (the immigrants) adapts to the previous stabilized situation. On the other hand, morphogenesis appears when the new structure, after the introduction of the third population, is dramatically different from the original stabilization between the two populations. In this article, we are particularly interested in this case.

## Results

### The three individual based Sakoda model

The Sakoda model consists of a two-dimensional lattice with $N = L\times L$ sites. Each site is characterized by a discrete variable, $x_{k}$, that may take a “colored” value in the set: empty, red, blue, and green, i.e.,  . When a node *k* is empty $(x_{k}=0)$ and only in this situation, another individual  , or  , or  may occupy the empty site and re-assign its own value. The lattice occupancy is arbitrarily provided initially with $N_1$ individuals of the type 1 (e.g., the “reds”  ) , $N_2$ individuals of the type 2 (the “blues”  ) and $N_3$ individual of type 3 (the “greens”  ). Finally, there are $N_0$ empty sites which we plot with a white empty space. Naturally, the sum of each occupancy states are the total available number of sites $N= \sum _{\sigma =0}^3 N_\sigma$.

The social interaction is mainly characterized by the possible attitudes among individuals which are summarized in a $3\times 3$ matrix, that we call the “*S*-matrix”, which has the form:The entries $s_{\alpha \beta }$ of the $S$-matrix take three possible values: $\{+1,0,-1\}$. These indicate, respectively, an attractive $(+1)$, a neutral (0), or a repulsive $(-1)$ attitude from members of the type $\alpha$ to the individuals of the type $\beta$.

In the original Sakoda model^7^, the preferences of an individual over a particular place in the lattice is based on the spatial locations of all other individuals and the attitudes among them. Following the original ideas of Sakoda, we propose a function that quantifies the social expectations of the individual *i*:

1 $$\begin{aligned} f_{i}(x_{i})=\sum _{k=1}^{N} J_{ik} \delta _S(x_{i},x_{k}), \end{aligned}$$

where $J_{ik} \ge 0$ denotes a symmetric ($J_{ik}=J_{ki}$) interaction strength. We avoid self-interactions by taking $J_{ii}=0$. On the other hand, the sign (repulsive, attractive or neutral) of the interaction is given by:

2 $$\begin{aligned} \delta _S(x_{i},x_{k})= & {} \left\{ \begin{array}{l l} s_{\alpha \beta } &{} \quad \text {for }x_{i}=\alpha \ne 0 \,\, \& \,\, x_{k} = \beta \ne 0 \\ 0 &{} \quad \text {otherwise} \end{array} \right. . \end{aligned}$$

We underline that, though $J_{ik}$ is symmetric, the full interaction $J_{ik} \delta _S(x_{i},x_{k})$ is not necessarily symmetric, because $\delta _S(x_{i},x_{k})$ is not symmetric, i.e., $\delta _S(x_{i},x_{k})\ne \delta _S(x_{k},x_{i})$. The coefficients $J_{ik}$ may include short and long range interactions. The mobility of an individual could be also of long or short-range movement.

An individual located at the node *i* would move towards an empty node *j*, if $f_{i}(x_{i})< f_{j}(x_{i})$. Here $f_{j}(x_{i})$ represents the potential value (1) at the empty node, *j*, occupied by the individual $x_{i}$. As a general rule, an individual chooses, everywhere in the lattice, a place *j* that represents the highest possible value of the social expectation function $f_{j}(x_{i})$. In case of degeneracy, that is, if there are *n* nodes, $j_1,j_2,\dots j_n$, such that $f_{j_1}(x_{i})=f_{j_2}(x_{i})=\dots =f_{j_n}(x_{i})$, then, one of these is selected randomly with the same probability. In a short movement case, the mobility of the individual is restricted to its Moore neighborhood (the eight closest neighbors).

Summarizing, the movement of individuals occur as follows: During an iteration step, an individual is selected randomly. Then, she or he (hereafter, we refer to an individual by female gender) evaluates the potential function at every available empty site in her range of movement for a potential new location. Next, the individual selects the site with the highest potential expectation value (1) and then she moves to this site. If no site improves the potential function, then the individual holds on in place. Lastly, the algorithm is iterated until the system reaches a fixed point; otherwise, it runs indefinitely.

In Ref. ^15^, we have demonstrated an energy theorem, namely if the *S*-matrix is symmetric, then, the Potts-like energy function^17^:

3 $$\begin{aligned} E[x] =-\frac{1}{2}\sum _{i,k} J_{ik}\, \delta _S(x_{i},x_{k}), \end{aligned}$$

is a non increasing function after a movement resulting from the Sakoda algorithm, i.e., *E*[*x*] is a non-strict Lyapunov function, $E_\text{after} \le E_\text{before}$. This result is important because it restricts the time evolution of a configuration. In other words, if the interaction *S* is a symmetric matrix, then, because *E*[*x*] cannot increase, the dynamics should stop in finite time.

Besides the energy theorem, we underline that the Sakoda rule preserves the initial number of individuals of all types. Therefore, all $N_0$, $N_1$, $N_2$, and $N_3$ keep their initial value during the evolution. Hereafter, we quantify the occupancy via the fractions: $\phi _\sigma = {N_\sigma }/{N}$.

Finally, for the sake of simplicity, as in Ref.^15^, we restrict our central results only to the case of short-range interactions and long-range movements: An individual evaluates her preferences considering the Moore neighborhood, that is $J_{ik}=1$ if *k* is in the eight closest neighbors of the site *i*, and $J_{ik}=0$ elsewhere. Then, the individual may look forward to the highest expectation at an empty node in the lattice.

### Migration induced social morphostasis–morphogenesis transition

We aim to study the robustness of the social structures under perturbation induced by a third population. As mentioned, we are interested in a manifestation of the Archer’s morphogenetic scheme^16^ under conditions of a sudden immigration of a third population into stabilized social structures. We analyze this by using computational simulations based on the Sakoda model of social interactions.

To study a morphogenetic transition we assume the existence of a stable social structure, then we add a fraction of immigrants with given attitudes or interactions. The further evolution would eventually determine if the new social structure is dramatically different from the original one, that is, if we either observe a morphogenetic cycle or we are in presence of a morphostatic cycle where immigrants adapt to the prevailing social structure.

We proceed as follows: we start with an initial state with two different populations of ( , ) with  that are randomly and uniformly distributed in the lattice (see Fig. 1a). Next, we run the Sakoda algorithm described in the previous section.

Figure 1b presents a Segregation social structure (S1), obtained for the the following interactions:  . After about $10^{5}$ steps the system reaches a stationary state (an attractor) (see Fig. 1b). Similarly, Fig. 2b shows the same protocol for the *Social workers* (S5) social structure ( ). The same procedure can be repeated for other social structures, however, in this paper we present the morphostasis-morphogenesis transition in the cases of these two social structures: S1 and S5. We select Sakoda’s segregation and social workers structures since they are relevant starting points for considering immigration effects. Segregation means attraction among individuals of the same group and repulsion to members of other groups; social workers, on the other hand, resembles a situation in which one group have a positive attitude toward others who in turn reject interaction. In this sense, immigrants arrive either to a rather hostile society in which segregation attitudes prevail, or to a society in which one group could be a friendly host (the social workers). Assuming these initial situations, we observe our results. Notice that S1 and S5 are different in one aspect in the segregation pattern, both the blue and red populations interact symmetrically reaching stable red and blue domains (see Fig. 1b). On the other hand, the case of S5 does not represent a symmetric interaction, thus the system does not reach an attractor, and more important, the final structure is not symmetric. Indeed, as it can be seen in Fig. 2b, the “red” individuals present a cohesive and stable community, but the “blue” ones do not form a coherent structure, because they are delocalized and moving all time around the empty sites.

At this point the immigration starts, we model it by adding by hand a randomly distributed “green” population with the same concentration as the blues, and reds (in the simulations  ). This can be seen in Fig. 1c. Then we define the interactions among the reds and the greens, as well as among the green with the blues, and the greens with themselves. From the $3^5$ possible cases, we restrict our work to 4 different cases that may be summarized with the following particular interaction matrix:4The  take the values of the desired social structure, and both $\sigma _1=\pm 1$ and $\sigma _2=\pm 1$ accept positively an immigrant ($\sigma _1=\sigma _2=1$), and the case of a rejected one ($\sigma _1=\sigma _2=-1$). The other two cases correspond to mixed situations. Notice that we have restricted our numerical analysis to the situation in which the immigrants attract themselves, that is  .

Figures 1 and 2 show evidence of the morphogenesis/morphostasis behavior of two well established social structures (S1 and S5). In particular, Figs. 1**d**,**g** exhibit two examples of the morphostasis process in which the immigrants,  , are forced to adapt to the previous structure (formed by  and  ). One notices that the blue and red structures are both stable and their original shape does not change its main characteristic. Figure 1**e**,**f** show examples of morphogenesis in which a third population  modifies the previous well established social structure of *Segregation* (S1).

One notices that in the case of symmetric interactions (Fig. 1**d**,**g**) the “greens” interact in two distinct manners: though, in Fig. 1**d** he “greens” are accepted by the “blues” and “reds”, they are not able to migrate inside the close domains of occupied by the “blues” and the “reds”. Hence, the most expected location for them is at the “blues”–“reds” interface domains. This is, for example, the case when immigrants occupy job positions that locals do not want to execute anymore—mostly low-income jobs. Immigrants are accepted as long as they accomplish a role which is hardly covered (or not covered at all) in society. This could be the situation in affluent societies in which locals ascend in social stratification, thereby rejecting unattractive jobs which are accepted by immigrants. To that extent, social structure remains the same (morphostasis), as the lower positions in social stratification do not disappear but are occupied by the newcomers. On the other hand, in Fig. 1**g**, the “greens” are rejected by the “blues” and the “reds”, therefore the most favorable configuration is total segregation, that is no one overlap the others, as seen in Fig. 1**g**. In this case, immigrants develop ‘their own world’—as it were. They do not change the fundamental structure of segregation in society, but add another layer to it. This is similar to a situation of immigration in the 19th century, as Europeans are invited by Latin American governments to colonize virgin territories where a segregation prevails between national locals and indigenous people. Immigrants do not change segregation between them (morphostasis) but rather fill empty spaces, developing their own schools, churches, and alike.

From a more analytic point of view, one notices that in these cases the dynamics stop in finite time, because the interactions are symmetric. Accordingly, the “green” immigrants have no time to modify the original social structure. On the contrary, the cases of Fig. 1**e**,**f** correspond to asymmetric interactions, hence the dynamics never stops, allowing time to the immigrants for changes in the original social structure. Here we speak of morphogenesis. These are cases in which immigrants play either a bridging or and intervening role between segregated people, thereby dissolving old social relations and recomposing it into new ones. This could be the case of a humanitarian intervention into a country affected by civil war, for example, or the allied intervention in Germany after World War II to deconstruct the segregation between German people and Jews.

For the case in Fig. 2, the interaction is never symmetric, hence the dynamics never stops and all individuals play forever in the social checkerboard, therefore, the pattern robustness may be tested in the long run. Notably, as before, the Fig. 2**g** shows a morphostasis behavior, while Fig. 2**e**,**f** reveal a morphogenetic behavior of the afore-established social structure of *Social-workers* (S5), thereby transforming the previous coherent society installed by the reds. In these cases, immigrants interact so strongly with the friendly hosts (the “reds”), that they become intertwined with them, allowing for morphogenesis. This is, for example, the case of “mestizo”-like structures. Additionally, though the patterns of Fig. 2**d**,**g** do not appear as the original structure Fig. 2**b**, the red structure mainly survives immigration in both cases. The non-coherent delocalized dynamics of the blue individuals is not transformed either. This could be the case in which immigrants relate to a particular stratum of the “reds”, commonly the lower stratum, while the upper red stratum remains immune to crossbreeding. Depending on the interactions, the green individuals may set-up a structure, as in Fig. 2**g**, even in the case whenever the greens are rejected by the two other populations. In the case that the greens are accepted, nor the greens neither the blues are allowed to set up a coherent structure, hence their population fluctuate around the red structure. Therefore, in both situations, we obtain a morphostasis.

A similar analysis may be pursed to all other social structures. Other cases reveal a similar behavior. A robust situation occurs whenever the immigrants are rejected by the former populations (the “blues” and “reds” individuals), that is whenever the interaction matrix correspond to $\sigma _1=\sigma _2=-1$ in the interaction *S*-matrix (4). In all these cases, the immigrants form coherent green structures inside the former structure already discussed in Ref.^15^. Essentially, the final configurations keep the green individuals separated from reds and blues. Moreover, there is a tendency of the “greens” to form closed domains. In the opposite case, wherever the immigrants are liked by the former structure, that is, for the following interaction: $\sigma _1=\sigma _2=1$ in (4), one has a different structural attractors. Generally speaking, the immigrants are placed on the boundaries of the pre-established structures. The inclusion of the “greens” does not modify dramatically the pre-established attractors. Therefore, these situations present a morphostatic behaviour. The general observed principle the morphostatic behaviour is observed in the case of symmetric interactions of the included immigrant respect to the former two individuals (Fig. 1**d**,**g**.

On the contrary, in the case whenever the interactions are not symmetric one observes in most cases that a morphogenetic phase dominates—as in the case of Fig. 2**e**,**f**. The exceptions are the social structures S7, S9 and S10, which correspond to a situation where the interactions are:

$$\begin{aligned} S_7= \left( \begin{array}{ccc} -1&{} 1 &{} \sigma _1\\ 1 &{} -1&{} \sigma _1\\ -\sigma _1&{}- \sigma _1 &{} 1 \end{array} \right) , \quad S_9= \left( \begin{array}{ccc} -1&{} -1 &{} \sigma _1\\ -1 &{} -1&{} \sigma _1\\ -\sigma _1&{}- \sigma _1 &{} 1 \end{array} \right) , \quad \& \quad S_{10}= \left( \begin{array}{ccc} -1&{} 1 &{} \sigma _1\\ -1 &{} -1&{} \sigma _1\\ -\sigma _1&{}- \sigma _1 &{} 1 \end{array} \right) . \end{aligned}$$

Here one notices at least in S9 and S10 an essential repulsive (negative) interaction. In these cases, the original structure, at least in S9 and S10, is mostly a random field with blues and reds separated by as much distance as possible between them. The inclusion of the greens does not really modify this pattern, displaying a random distribution of “reds”, “blues” and “greens”.

As we shall see next, a quantitative description of a morphostasis-morphogenesis transition can be done by using the Potts-like energy (3). An interesting open question concerns the relation of this “energy” with real social parameters obtained by social scientist with the methods of questionaries, surveys, and field measurements.

### Energy criteria for morphostasis–morphogenesis transition

**Figure 3:**
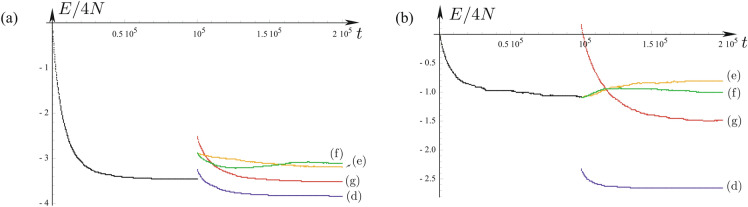
Evolution of the energy as function of time for previous simulations: (**a**) Fig. 1 and (**b**) Fig. 2. The initial time ($t=0$) corresponds to the step (**a**) in previous figures. The “blues” and “reds” individuals interact up to time $t=10^5$, as it can be seen, the energy (blacks points) decreases. The “greens” migrate randomly at $10^5$, and then we run the four different interactions (**d**–**g**) to establish a new social structure.

Qualitatively, we notice that morphostasis occurs for symmetric interactions of the migration population, while morphogenesis happens for non-symmetric interactions of the third population. A more quantitative approach can be done on the basis of the energy (3). As shown in Ref.^15^, the energy mainly decreases (and necessarily decreases if the interaction *S*-matrix is symmetric) as time passes by. Hence, it provides an excellent tool for the study of the “potential” evolution of the system. The evolution of the energy (3) is plotted in the next Fig. 3 for the previous two cases (S1) and (S5) already shown in Figs. 1 and 2.

In both plots for $t\in [0,10^5]$, the Sakoda algorithm runs only with the “blue” and “red” individuals. Although the case of (S5) does not have a symmetric interaction, as it can be readily seen in Fig. 3, both energies decrease mainly in time, independently of the symmetry of the interactions. This has been often observed in Sakoda’s^15^ and Schelling’s^18^ models as well as. At $t=10^5$, the “greens” are added randomly, then for $t\in [10^5,2\times 10^5]$ the algorithm runs with the three distinct populations. One notices that the observed energy decreasing principle does not hold anymore.

This landmark peculiarity indicates a probable criterion for the morphogenesis/morphostasis transition. Indeed, the energy decreases for the four cases of morphostasis (Figs. 1d,g, 2d,g), while the energy increases in three of the four cases of morphogenesis (Figs. 1**f**, 2**e**,**f**) The case of Fig. 1**e** presents a peculiarity: the energy decreases as the third population is included, however one notices apparent major changes in the new social structure. In conclusion, we may conjecture that, in most cases, for morphostasis the Sakoda energy decreases, while morphogenesis correlates with an anomalous increasing energy. Sociologically speaking, more effort is required from agents to change social structures than to maintain them.

## Discussion

In this paper, we develop a generalization of Sakoda’s model for three populations to study the dynamics and social consequences of immigration phenomena on social structures. The main tenet of Sakoda’s approach is to reproduce agent dynamics according to a finite set of rules of repulsion, attraction, or indifference. In particular, we approach migrations from a perturbative point of view: given an established social structure, we studied what happens when a third population is introduced into an artificial society, in this case, into an in silico simple society.

The most important finding of this work is the interpretation of immigration dynamics from Archer’s sociological approach in connection with Sakoda’s social structures. In this sense, the third population either dramatically modifies the previous society (morphogenesis), or new individuals adapt to the previously established social structure (morphostasis). Even several new social structures may appear because of the vast number of possibilities given by the multiple configurations of the Sakoda interactions. We notice that morphostasis occurs for symmetric interactions of the migration population, while morphogenesis appears for non-symmetric interactions of the third population. This is an important finding, for sociologically symmetric interactions take place mostly among people who consider themselves as equals (similar consideration regarding social positions, e.g. professionals, scholars); while asymmetric interactions unfold in hierarchical or confrontational contexts (people with different educational background or from different social positions or ethnic groups). This means that the social structures in host society are most likely to change (morphogenesis) when dissimilar social groups meet; while society structurally remains (morphostasis) when groups are rather similar in social composition.

This seems to be described quantitatively by the energy principle shown in equation (3), providing a descriptor of the evolution of the system, at least, for the two representative cases studied in this work: segregation (S1) and social workers (S5). We also note that, in a general manner, the energy decreases for morphostasis cases (Figs. 1d,g, 2d,g), while the energy increases for morphogenesis cases (Figs. 1**e**,**f**, 2**e**,**f**), which drives us to make a conjecture of the morphostasis/morphogenesis anomalous decreasing/increasing energy relation, respectively. This could be a general sociological measure of the effort that society devotes to maintain or change its structures.

A natural question that arises from this work is the variation of the network topology. Changing the grid configuration and neighborhood relations range might be explored, for example, a most general framework that opens the possibility of the use of complex networks. There are several works that consider segregation dynamics in complex networks^19–22^. However, in the context of migrations, Sakoda’s model may bring a wider perspective that may be considered in urban planning and policy interventions^23^. Moreover, a generalization of the energy principle in complex networks, where the connectivity varies for each node may bring interesting insights from the study of physical systems.

The approach we present in this article might be used in a variety of social research fields. For example, in recent years, increasing interest arises in the field of pre-historic and native human interactions, where breakthroughs related to social interactions among different human species and native populations have brought diverse interesting results^24–27^. In this sense, simulating the morphogenetic dynamics of different populations may shed light on many hypotheses regarding pre-historic migrations or social events with morphogenetic dynamics information. Other extended additions of this model, like considering violent acts, casualties, etc., in the simulations like decreasing-increasing populations sizes may help complement different studies in social sciences.

## Methods

### Proof of the energy principle

Consider the Potts-like energy (3), with a symmetric *S*-matrix. Therefore, $\delta _S(x_{j},x_{k})=\delta _S(x_{k},x_{j})$ for two arbitrary sites *j* and *k* in the lattice. Then, consider the exchange of sites *l* (occupied) and *q* (empty), *i.e.*, $x_{l}=\pm 1$ and $x_{q}=0$. Hence, the initial configuration and the subsequent configuration after swap between the sites *q* and *l* are:

$$\begin{aligned} x=\{\dots , x_{l},\dots ,x_{q}=0,\dots \}, \quad x'=\{\dots ,x_{q}=0,\dots ,x_{l}, \dots \}. \end{aligned}$$

Next, if the *S*-matrix is symmetric, then the energy difference between the initial and the actual configuration becomes

5 $$\begin{aligned} \Delta E\,=\, & {} E[x']-E[x]=\sum _{j\ne q,l}\left( J_{lj}-J_{qj}\right) \delta _S(x_{l},x_{j}). \end{aligned}$$

Here we have used $J_{jj}\delta _S(x_{j},x_{j})=0$, because we imposed that $J_{jj}=0$ and $\delta _S(x_{q},x_{j})=0$ for all *j*, since the $\delta _S$ function evaluated at a vacancy state $x_q=0$ vanishes. Next, the intrinsic mechanism of Sakoda’s rule imposes that the local value of (1), $f_l$, of the individual $x_{l}$, is smaller than the interaction at the site *q*, *i.e.*, $f_{q}(x_{l}) > f_{l}(x_{l})$ :

6 $$\begin{aligned} f_{q}(x_{l})=\sum _{j}J_{qj}\delta _S(x_{l},x_{j}) > f_{l}(x_{l})=\sum _{j}J_{lj}\delta _S(x_{l},x_{j}). \end{aligned}$$

Therefore, re-writing the r.h.s. of (5) in terms of the expressions $f_q$ and $f_l$, previously defined in Eqs. (6) and (1) respectively, one obtains:

7 $$\begin{aligned} \Delta E = f_{l}(x_{l})-f_{q}(x_{l}) \le 0. \end{aligned}$$

In conclusion, if *S* is a symmetric matrix, then after a swap between the configurations *x* and $x'$, $\Delta E \equiv E[x']-E[x] \le 0$, that is, the energy (3) is a decreasing quantity after a movement.
